# Hybrid Reverse Molecular Dynamics Simulation as New Approach to Determination of Carbon Nanostructure of Carbon Blacks

**DOI:** 10.1038/s41598-020-60372-0

**Published:** 2020-02-27

**Authors:** Masaya Ishida, Tomonori Ohba

**Affiliations:** Graduate School of Science, Chiba University, 1-33 Yayoi, Inage, Chiba, 263-8522 Japan

**Keywords:** Chemistry, Method development, Nanoscale materials, Nanoparticles, Structural properties

## Abstract

Various carbon materials have been fabricated for use as catalyst supports, carriers, adsorbents, and electrodes as well as in other advanced applications. The performances of carbon materials in such applications can be improved by adjusting their physical properties, especially their nanostructures. The determination of the carbon nanostructure is thus considerably important. Reverse Monte Carlo and hybrid reverse Monte Carlo simulations, which are used to analyze the diffraction patterns of carbon materials, can be used to obtain nanostructure images. Here, we describe a new approach to carbon nanostructure investigation, namely, hybrid reverse molecular dynamics (HRMD) simulation. This approach has the advantage that all of the carbon atoms move toward probable carbon structures by force fields to adapt a simulated diffraction pattern to an experimental one, in contrast to the random movements in reverse Monte Carlo and hybrid reverse Monte Carlo simulations. HRMD simulation also prevents the formation of inappropriate structures.

## Introduction

Carbon structures have been evaluated using various techniques such as X-ray diffraction (XRD), electron and neutron diffractions, light, electron, and X-ray spectroscopies, light and electron microscopies, and adsorption techniques. XRD analysis on carbon materials is commonly used for obtaining the crystal structures, including the interlayer and intralayer distances as well as the crystallite size^1–3^. The G, D, and 2D bands in Raman spectra provide information on the carbon microstructure^4,5^. A combination of XRD and Raman techniques is often used, and they provide complementary and comprehensive information^6^. Microscopic techniques, especially transmission electron microscopy (TEM), are also useful because they enable the direct observation of structures at the nano- and microscales^7^. TEM observations are especially essential for evaluation of the structures of nanocarbon materials. The surface area and pore structures can be determined on the basis of the adsorption of N_2_, CO_2_, and other gases. Quantum calculations, structural simulations, and molecular simulations are becoming powerful tools for understanding the experimental results obtained by various spectroscopic, microscopic, and adsorption techniques on their own and in association with experimental analyses^8–10^. Structural simulations and molecular simulations, in particular, have the advantage of providing clear images of the nanostructures of carbon materials. Although XRD is one of the most powerful tools for investigating the crystalline structure among these techniques, carbon materials are mainly amorphous or nanocrystalline, and thus, little structural information can be obtained from XRD analysis.

A combination of XRD and simulation techniques has been used to compensate for the difficulties associated with direct XRD analysis. Reverse Monte Carlo simulation was first used to investigate amorphous structures by McGreevy and Pusztai^11^. Radial distribution functions (or structure factors) are normally one-dimensional and obtained experimentally by X-ray or neutron diffraction. In reverse Monte Carlo simulation, the three-dimensional structure is evaluated by fitting a simulated radial distribution function to an experimental radial distribution function. This technique was first applied to liquid argon, but it is applicable to various materials. Pusztai and coworkers used this technique to investigate colloids^12^. The reverse Monte Carlo technique has also been used to evaluate the structures of carbon materials^13,14^. Snapshots of porous carbons were obtained by reverse Monte Carlo simulation, and then, the structures were further investigated by TEM and adsorption^15,16^.

Reverse Monte Carlo simulation has the problem that highly strained and unstable structures appear. For carbon structural evaluation, potential calculations have been incorporated into the reverse Monte Carlo technique; this is known as hybrid reverse Monte Carlo (HRMC) simulation^17,18^. Palmer and Gubbins evaluated the validity of reverse Monte Carlo and HRMC simulations by comparing the results with the structural data obtained from quenched molecular dynamics (MD) simulations^19,20^. Similar radial distribution patterns were obtained with the reverse Monte Carlo and HRMC techniques, but the nanostructures were different. The reverse Monte Carlo simulation gave unrealistic structures, whereas HRMC provided images that were qualitatively similar to the target structure obtained by quenched MD simulation. The HRMC simulation produced a physically reasonable nanostructure model. Nguyen and coworkers examined the fluid properties of porous carbons modeled by HRMC simulation^21,22^. Analytical techniques associated with HRMC simulations have also been developed for identifying amorphous carbon structures^23^. A ring-clustering algorithm associated with HRMC simulation was developed to obtain ring connectivity to enable images of the graphene layer structure to be obtained^24^. Further developments in modeling and analytical techniques based on well-fitting experimental data are needed to understand complex carbon structures.

Reverse Monte Carlo simulation and HRMC simulation minimize the discrepancy between simulated and experimental diffraction patterns or radial distribution functions. The Monte Carlo algorithm in those simulations has the inherent problem of a finite chance that an atom is trapped in a molecular group and rarely exchanged into a neighboring group by metastable bond formation in the Monte Carlo scheme and the structure factor in the reverse Monte Carlo scheme. This results in a small acceptance ratio in displacement steps, even in a fluid system without interatomic bonds. The displacement of a carbon atom is slightly accepted, and a carbon atom hardly moves in a reverse Monte Carlo simulation because a carbon material contains many C–C bonds. Tóth and Baranyai proposed a reverse molecular dynamics (RMD) simulation to overcome this disadvantage^25^. The RMD simulation has the advantage that an atom or a molecule is less trapped in a local configuration minimum and the pseudo-dynamics, which are not the actual dynamics, approach the equipartition state. Another advantage is that multithread programming in the RMD is easily achieved, the same as in a typical MD simulation. The validity of the RMD simulation was examined by comparison with the reverse Monte Carlo simulation; the simulations provided equivalent results to experimental ones^25^. The reverse Monte Carlo scheme is simpler and more economical for simple atomic systems, but for large and/or complicated systems such as molecular systems with interatomic bonds, the RMD simulation has advantages. However, the RMD simulation has a problem similar to that of the reverse Monte Carlo simulation. We therefore developed a new technique for the analysis of diffraction data, namely, hybrid reverse molecular dynamics (HRMD) simulation, and used it for the XRD analysis of various types of carbon black as standard carbon materials. The HRMD simulation technique can minimize the discrepancy between experimental and simulated radial distribution functions by the RMD scheme and the total energy by MD scheme. The structural parameters obtained from HRMD simulations were compared with experimental values.

## Results

**Figure 1:**
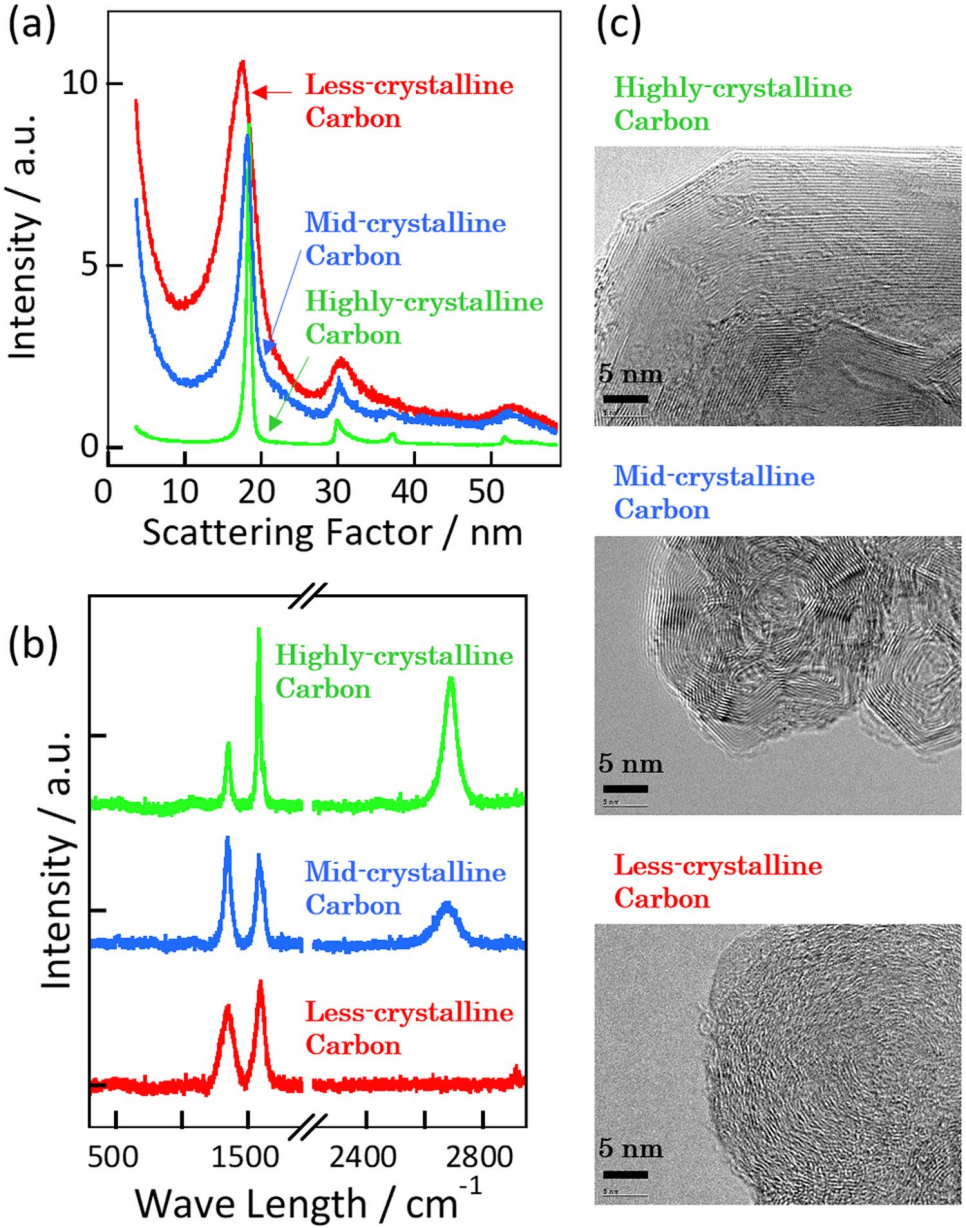
Experimentally determined structures of three carbon materials, i.e., highly crystalline carbon (#4040b), mid-crystalline carbon (Denka Black), and less-crystalline carbon (#32b): (**a**) XRD patterns, (**b**) Raman spectra, and (**c**) TEM images.

The peak widths in the XRD patterns in Fig. 1a indicated that the crystallinity of carbon black #32b was low and that of #4040b was highly crystalline. The crystal structure of Denka Black was between those of #32b and #4040b. Carbon black #32b, Denka Black, and #4040b are therefore referred to as less-crystalline, mid-crystalline, and highly crystalline carbons, respectively. The distinct peaks around the scattering factors *s* = 17–18, 30, and 51–53 nm^−1^ are attributed to the (002), (10), and (11) faces, respectively. Scattering factor *s* here is defined as 4πsin(θ)/λ. The XRD peaks at 36 nm^−1^ from the (004) face were also observed for the mid-crystalline and highly crystalline carbons, but they were hardly visible for the less-crystalline carbon. The crystallite sizes for the (002) and (10) directions were 5.0 and 5.3 nm for the less-crystalline carbon, 11 and 16 nm for the mid-crystalline carbon, and 37 and 33 nm for the highly crystalline carbon. The G/D band intensity ratios in the Raman spectra in Fig. 1b also show that the crystallinity of the highly crystalline carbon was higher than those of the others; the values were 2.8, 0.8, and 1.4 for the highly, mid-, and less-crystalline carbons, respectively. The D and G bands appeared at approximately 1350 and 1600 cm^−1^, respectively. The D band for the less-crystalline carbon was broader than that for the mid-crystalline carbon, although the G/D band intensity ratio was higher. The full width at half maximum values of the D bands were 40, 60, and 120 cm^−1^ for the highly, mid-, and less-crystalline carbons, respectively. The broad D band is a result of a disordered distribution of point defects, and the broadening of the D bands was consistent with the XRD analyses, although the G/D intensity ratio for the mid-crystalline carbon was smaller than that for the less-crystalline carbon. This suggested that mid-crystalline carbon had higher crystallinity than the less-crystalline carbon from the XRD analyses and the broadening of the D bands, while the G/D intensity ratios proposed more disorder for the mid-crystalline carbon. The TEM images in Fig. 1c clearly show these crystallinities; the highly crystalline carbon consisted of uniformly stacked graphene layers larger than 10 nm, whereas the less-crystalline carbon consisted of randomly stacked graphene layers smaller than 3 nm. The graphene layer size in the mid-crystalline carbon was 5–10 nm, i.e., between those of the less- and highly crystalline carbons. The scanning electron microscopy images in Fig. S1 indicated that all of the carbon samples consisted of submicroscale particles with diameters less than 100 nm.

**Figure 2:**
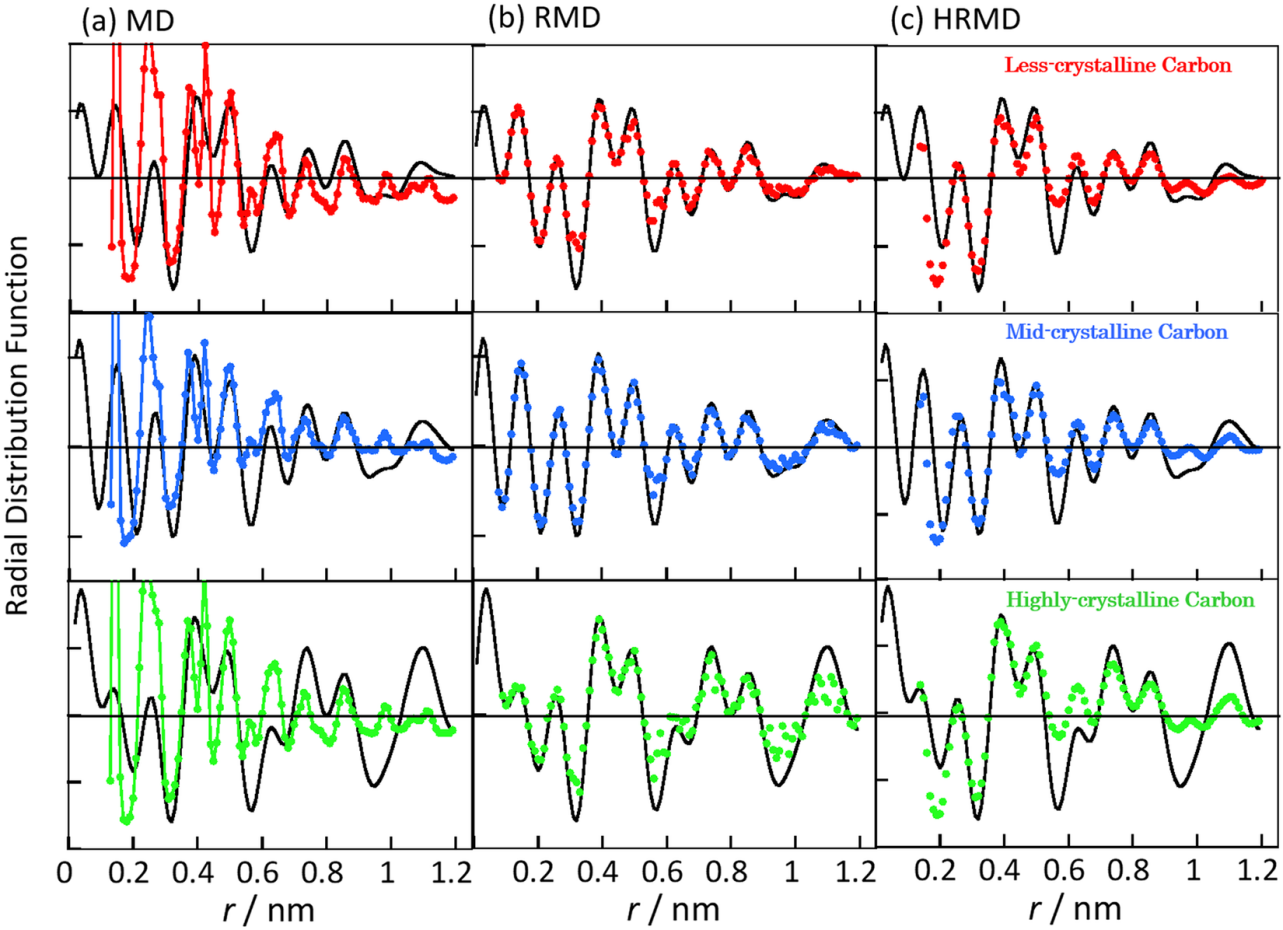
Radial distribution functions calculated from MD (**a**), RMD (**b**), and HRMD simulations (**c**). Red, blue, and green curves represent simulated radial distribution functions for less-, mid-, and highly crystalline carbons, respectively. Electron radial distribution functions (black curves) obtained experimentally by XRD are included for comparison.

**Figure 3:**
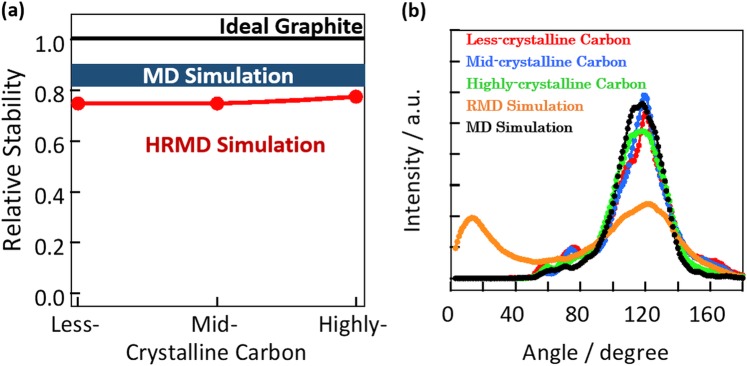
Stabilized structures of carbon black. (**a**) Relative stabilization energies of less-, mid-, and highly crystalline carbons (red). Relative stabilized energies of carbons at a density 1.0–2.0 g cm^−3^ and ideal graphite are shown by the blue region and black line, respectively. (**b**) Bond angle distributions of less- (red), mid- (blue), and highly crystalline carbons (green) in HRMD simulation. Bond angle distributions of highly crystalline carbons in RMD simulation (orange) and MD simulation (black) are for comparison.

Figure 2 shows the simulated radial distribution functions of highly, mid-, and less-crystalline carbons in the MD, RMD, and HRMD simulations as well as the corresponding electron radial distribution functions. The radial distribution functions in the MD simulations at each density of carbon black had relatively sharp peaks, especially the first peak from the honeycomb structure of graphite; these differ from the electron radial distribution functions. Carbon black samples had graphitic structures, and those peak positions in MD simulations roughly agreed with experiments. The peak intensity ratios were however much different. The radial distribution functions obtained in the RMD simulations were perfectly in agreement with the experimental ones. The HRMD simulations also gave good agreement with the experimental ones, although the identity was worse than in the RMD simulations. The XRD patterns in RMD and HRMD simulations are also good agreement with the experimental ones, as shown in Fig. S2. This relationship between the HRMD and RMD simulations is similar to that between hybrid reverse Monte Carlo and reverse Monte Carlo simulations^17^. However, the carbon structures in the RMD simulation were unstable, as described below. Figure 3 shows the stabilized energies of carbons and bond angle distributions in the HRMD simulations. The relative stability is defined as the stabilized energy in a system divided by the stabilized energy of ideal graphite. The stabilized energies in the MD simulations of carbons at 1.0–2.0 g cm^−3^ were between 80% and 90% of that of ideal graphite, as shown in Fig. 3a. The relative stabilized energies in the RMD simulations were approximately −10^5^, which means that the structures were rather unstable. The snapshots of the highly crystalline carbon in Fig. S3 indicated that many three and four carbon rings were formed, and carbon atoms were partially overlapped, although the simulated radial distribution functions in the RMD simulation agreed perfectly with the experimental ones. In the HRMD simulations, the stabilized energies were 75%, 75%, and 78% for the less-, mid-, and highly crystalline carbons, respectively. The bond angle distributions of C–C–C in the HRMD simulations in Fig. 3b had the main peak at 120 degrees, indicating six ring structure, and the small shoulder peaks around 60 and 70 degrees, originating from trigonal and pentagonal ring structures, respectively. On the other hand, the bond angle distributions in the MD and RMD simulations had only the relatively sharp six ring peak, and broad six ring and unrealistic small angle peaks, respectively. The unrealistic peak in the RMD simulation became the major weakness of structure evaluation in RMD simulations. Those results indicate that the HRMD simulation technique for analyzing carbon structures is satisfactory in terms of both the radial distribution function and the stabilized energy. The calculated structures are therefore potentially realistic. The stabilized energies of the carbons indicated that, as expected, the stability increased slightly with the increase in crystallinity, as observed from the XRD patterns and Raman spectra in Fig. 1.

**Figure 4:**
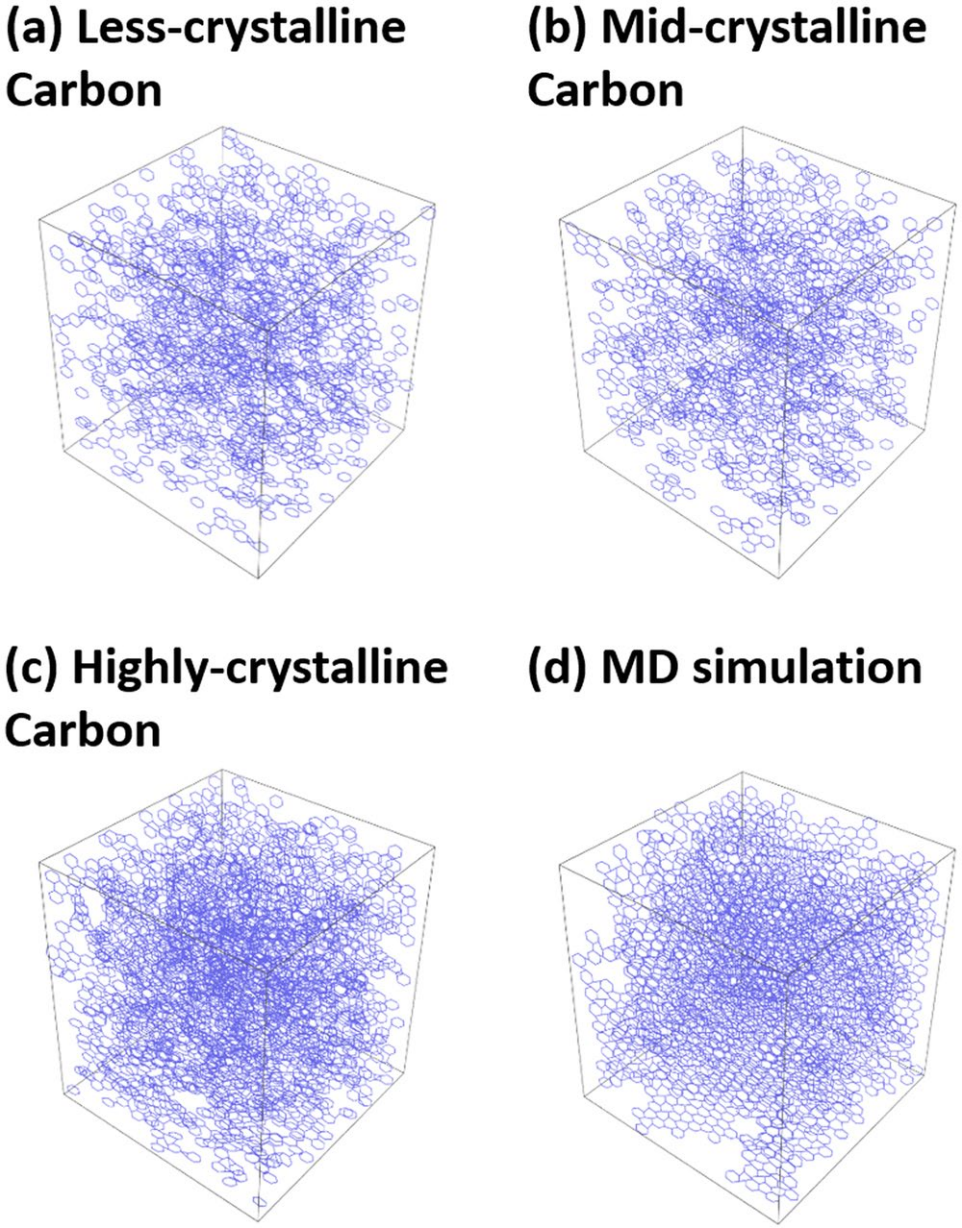
Snapshots obtained from HRMD simulations of less- (**a**), mid- (**b**), and highly crystalline carbons (**c**), and the MD simulation of carbon at 1.5 g cm^−3^ (**d**).

**Figure 5:**
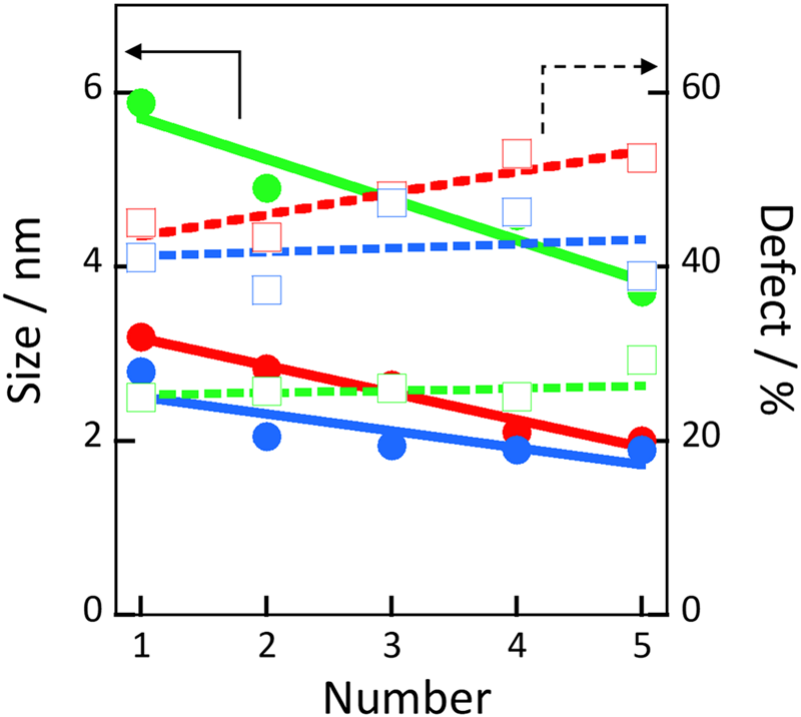
Average graphene unit sizes (solid lines) and defective structure ratios, including edge carbons (dashed lines), as a function of the graphene unit number from largest to fifth-largest graphene size for less- (red), mid- (blue), and highly crystalline (green) carbons.

Figure 4 shows snapshots of the carbons after HRMD and MD simulations. The crystallinity order was from less- to highly crystalline carbon; the carbon structure in the MD simulation had the highest crystallinity. Benzene rings were partially bound with each other in the less-crystalline carbon, and they were well bound in the highly crystalline carbon and MD simulation. Benzene rings thus tend to align in a similar direction because of the formation of a graphene-like structure. Figures S4–S6 show the graphene units extracted from these snapshots. Carbon atoms with a C–C bond length less than 0.2 nm were here considered to be in the same graphene unit. Graphene layers were connected mainly at the edges of graphene. The average graphene unit size and number of defective structures were determined from these graphene units (Fig. 5). The graphene unit sizes were 2–3 nm for the less- and mid-crystalline carbons and 4–6 nm for the highly crystalline carbon. The values for the less-crystalline carbon were approximately half those of the values obtained by XRD analysis (Fig. 1a) and similar to the sizes obtained from the TEM image (Fig. 1c). The graphene unit size calculated in the HRMD simulation for the highly crystalline carbon was much smaller (one-half to one-fourth) than those obtained by XRD and TEM. As the simulation box size was 5 nm, the simulated graphene unit size of 4–6 nm was almost the maximum graphene unit size in the HRMD simulation, which is the reason for the smaller graphene unit than those obtained by XRD and TEM, i.e., amorphous and/or less-crystalline structures were preferentially evaluated because of the small simulation box, whereas XRD and TEM analyses tend to focus on crystalline structures intentionally and/or unintentionally. For example, sharp peaks of the XRD pattern by the crystalline structure screen broad weak peaks by the amorphous structure. The defective structure ratios for the less-, mid-, and highly crystalline carbons were 40–55%, 40–45%, and 25%, respectively; these are defined as the number of partly unbound carbon atoms divided by the total number of carbon atoms in a graphene unit. The defective structure ratio in the largest graphene unit for the less-crystalline carbon was similar to that for the mid-crystalline carbon. The defective structure ratio for the less-crystalline carbon increased more than others with decreasing graphene unit size. The information of various graphene units is rarely obtained from the other analysis techniques, and this analysis has the advantage of graphene structural analysis. As mentioned above, the D/G intensity ratios in the Raman spectra (Fig. 1b) were 0.68, 1.3, and 0.4 for highly, mid-, and less-crystalline carbons, respectively; the full width at half maximum values of D bands were 40, 60, and 120 cm^−1^, respectively; and the densities were 1.0, 0.9, and 1.5 g mL^−1^, respectively. The defective structure analysis by HRMD simulations indicated the same order as evaluated from the XRD analyses and the broadening of D bands in the Raman spectroscopy, but the order was different from that evaluated from the D/G intensity ratios in Raman spectroscopy. This is because the results from HRMD simulation are inherently influenced by XRD information.

**Figure 6:**
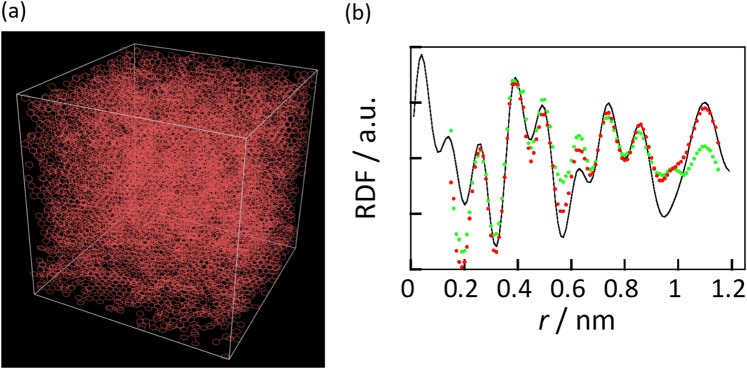
Structure of highly crystalline carbon using the larger unit cell. (**a**) Snapshot in HRMD simulation of highly-crystalline carbon using 8.0 × 8.0 × 8.0 nm^3^ unit cell. (**b**) Radial distribution functions in HRMD simulations using 8.0 × 8.0 × 8.0 nm^3^ unit cell (red) and 5.0 × 5.0 × 5.0 nm^3^ unit cell (green), and experiment (black) for the highly-crystalline carbon.

We developed a new approach to carbon structure determination by using HRMD simulation, which is a structural analysis technique that uses a combination of experimental XRD data and molecular dynamics simulations. The analytical techniques based on RMC and HRMC simulations have been previously reported. Those techniques have weakness regarding the random movement. On the other hand, HRMD simulation has the advantage that it allows for the simultaneous movement of all of the carbon atoms toward a more realistic structure. In addition, conventional analytical methods such as XRD and TEM tend to focus on the crystalline structure. However, HRMD simulations extract amorphous and/or less-crystalline structures from the whole carbon structure and give results in agreement with Raman spectroscopic results as well. This technique is useful because various physical parameters such as the graphene unit size and defects are obtained simultaneously. HRMD simulation can also be used for highly crystalline structures by using larger simulation boxes in the calculation. The result using the larger simulation box of 8.0 × 8.0 × 8.0 nm^3^ is shown in Fig. 6. The snapshot in the larger simulation box was similar to that in the smaller simulation box. The radial distribution function in the larger simulation box, however, had better fitting to the experimental one. This suggested that a simulation box with a size similar size to the crystallite size was useful for better analysis, but the nanostructure was rarely changed, even with the use of a small simulation box. This method will continue to be improved, and more accurate and efficient structural evaluations will become possible by combining it with other evaluation techniques.

## Methods

The HRMD simulation proposed here is a combination technique composed of an MD scheme and RMD scheme to obtain a more realistic structure satisfying both structure agreement with experimental parameters and a stable structure. Potential minimization in the MD scheme was performed to avoid formation of unrealistic physical structures. Interatomic interactions with bond angles and torsion affect the local bonding environment. We used the environment-dependent interatomic potential in an MD scheme, which is a potential function available, for a high-speed calculation^26,27^. The HRMD method is however independent of the potential model and another potential model is also available. In the RMD scheme, the discrepancy between the simulated and experimental radial distribution functions was calculated using the following equation:

1 $$\chi (r)={g}_{sim}(r)-{g}_{exp}(r)\times {w}_{int}$$

where *g* and *χ* are the radial distribution function at interatomic distance *r* and the difference between the simulated and experimental radial distribution functions. *w*_*int*_ is the weighting function for minimizing the difference between the simulated and experimental radial distribution functions. Radial-dependent forces for all the atoms were then calculated with a weighting function *w* in a RMD simulation:

2 $$F(r)=-\frac{\chi (r+\delta r)-\chi (r-\delta r)}{\delta r}\times w$$

These forces in the RMD and MD simulations were added into the HRMD simulations; the velocities and atomic positions were determined using the leapfrog Verlet algorithm with the heat-bath-coupling method.

The carbon densities were approximately 1.0, 0.9, and 1.5 g mL^−1^ in HRMD simulations of three carbon black samples, namely, #32b, Denka Black, and #4040b, respectively. A grand canonical ensemble was used in the MD scheme to maintain the densities. The conventional canonical ensemble MD simulation was periodically interrupted by a trial to create or remove a benzene unit. The probability was calculated as follows:

3 $${P}_{\pm }=exp[\mp \varDelta E/{k}_{B}T]+kd/{d}_{0}$$

where *P*_±_, *∆E*, *k*_b_, *T*, *k*, *d*, and *d*_0_ are the probabilities of benzene unit creation and removal, energy difference in a trial of creation or removal, Boltzmann constant, temperature, density adjustment coefficient, current density, and setting density, respectively. The creation and removal steps were performed according to the Metropolis sampling scheme with random insertion and random selection of a benzene molecule into the simulation box, respectively. The rotation of a newly created benzene unit was randomly set, while the velocity was set at zero. The overlap between newly created benzene and existing benzene units was rarely accepted, although the CC bond formation with favorable orientations was accepted.

The total pseudo-calculation time was more than 40 ps by an accumulation of time steps of 0.01–0.001 fs. The temperature was set to 300 K. The time step and temperature determine the migration length at a step, and a longer time step and higher temperature lead to faster equilibration, whereas it is easy to be unstable. An appropriate time step and temperature have to be chosen during calculations. Grand canonical MD simulations were first conducted to obtain the initial configuration in systems, and the simulations were passed on RMD or HRMD simulations when densities in the simulation boxes attained the target densities. The simulation box size was 5.0 × 5.0 × 5.0 nm^3^ for all of the RMD, HRMD, and MD simulations, and a periodic boundary condition was used in all three directions. Another simulation box 8.0 × 8.0 × 8.0 nm^3^ was also adopted for the highly crystalline carbon. The trial ratio for the RMD and MD schemes in the HRMD simulation also controlled the accumulated forces from the MD and RMD schemes. We performed the HRMD simulation as well as the RMD and MD simulations.

The nanostructures of three carbon black samples (#32b and #4040b, Mitsubishi Chemical Co., Tokyo, Japan, and Denka Black, Denka Co., Tokyo, Japan) were determined by HRMD simulations. The XRD patterns were recorded using Cu Kα radiation with a wavelength of λ = 0.154 nm at 40 kV and 40 mA (SmartLab, Rigaku Co., Tokyo, Japan). The crystal sizes were evaluated with the Scherrer equation. Raman spectra were recorded using a Nd:YAG laser at a power of 0.1 mW (NRS-3000; JASCO, Tokyo, Japan). TEM was used for direct observation of the nanostructures (JEM-2100F, JEOL Co., Tokyo, Japan).

### Data availability

The datasets generated during and/or analyzed during the current research are available from the corresponding author on reasonable request.

## Supplementary information

Supplementary Information.
