# Supplementary material for: Hybrid Reverse Molecular Dynamics Simulation as New Approach to Determination of Carbon Nanostructure of Carbon Blacks
Source: Sci Rep. 2020 Feb 27;10:3622. doi: 10.1038/s41598-020-60372-0 (PMC7046698; doi:10.1038/s41598-020-60372-0)
Supplement: Supplementary file 1 — Supplementary Information. [file 41598_2020_60372_MOESM1_ESM.pdf]

Supplementary Information

Hybrid Reverse Molecular Dynamics Simulation as New Approach to Determination of Carbon Nanostructure of Carbon Blacks

Masaya Ishida and Tomonori Ohba*

Graduate School of Science, Chiba University, 1-33 Yayoi, Inage, Chiba 263-8522, Japan

Figs. S1–S6

**Corresponding author*. E-mail address: ohba@chiba-u.jp (T. Ohba)

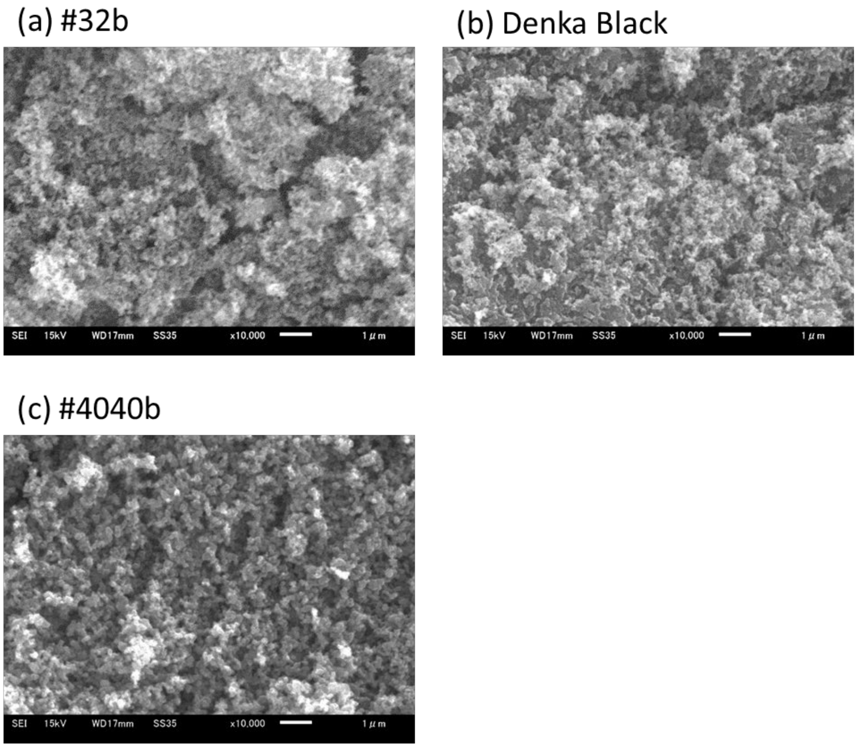

Fig. S1 SEM images of #32b (less-crystalline carbon), Denka Black (mid-crystalline carbon), and #4040b (highly-crystalline carbon).

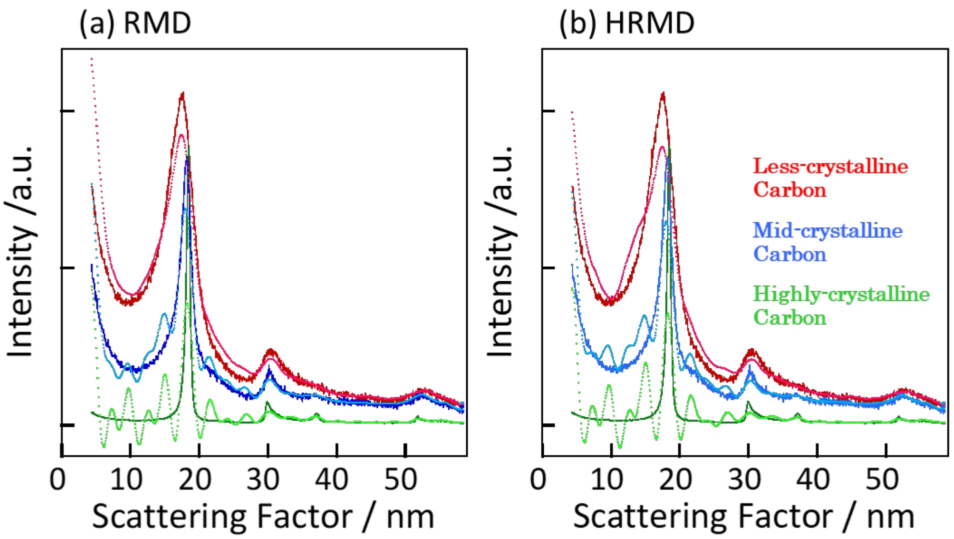

Fig. S2 XRD patterns obtained experimentally (dark-colored curves) and by simulations (light-colored curves) for less-crystalline carbon (red curves), mid-crystalline carbon (blue curves), and highly-crystalline carbon (green curves).

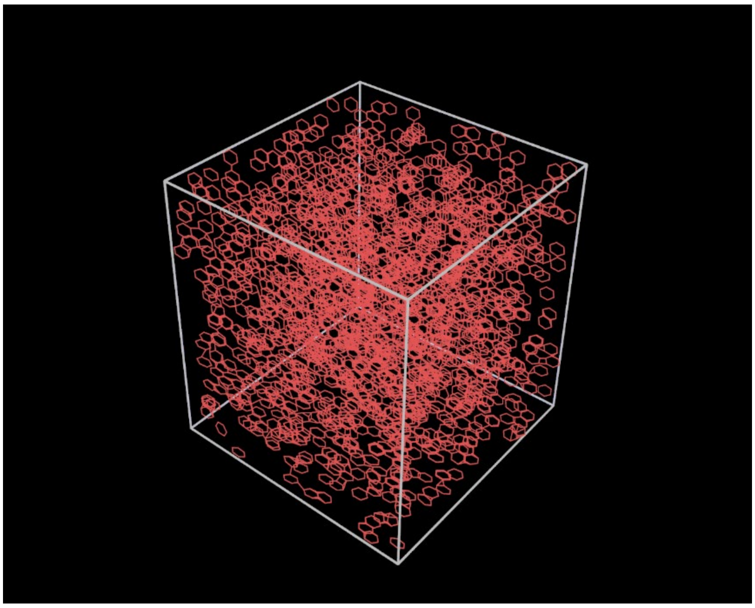

Fig. S3 Snapshot obtained from RMD simulation of highly-crystalline carbon.

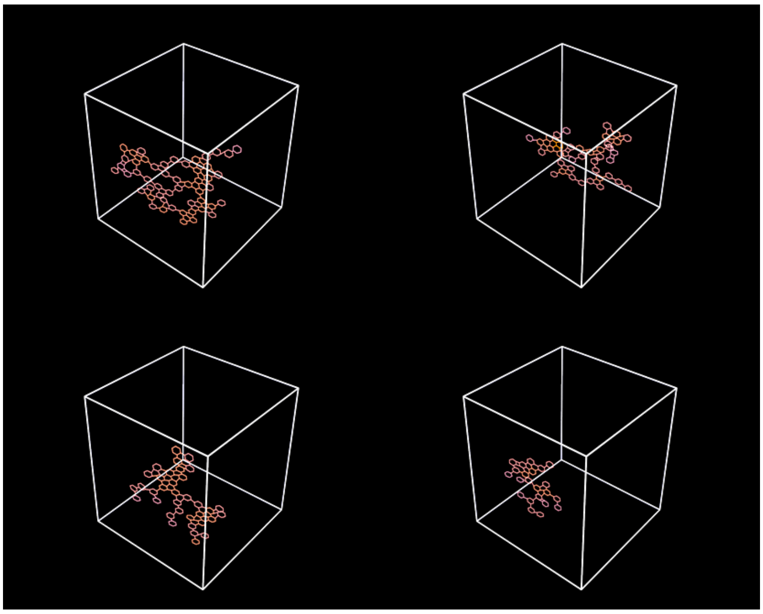

Fig. S4 Graphene units in HRMD simulation of less-crystalline carbon.

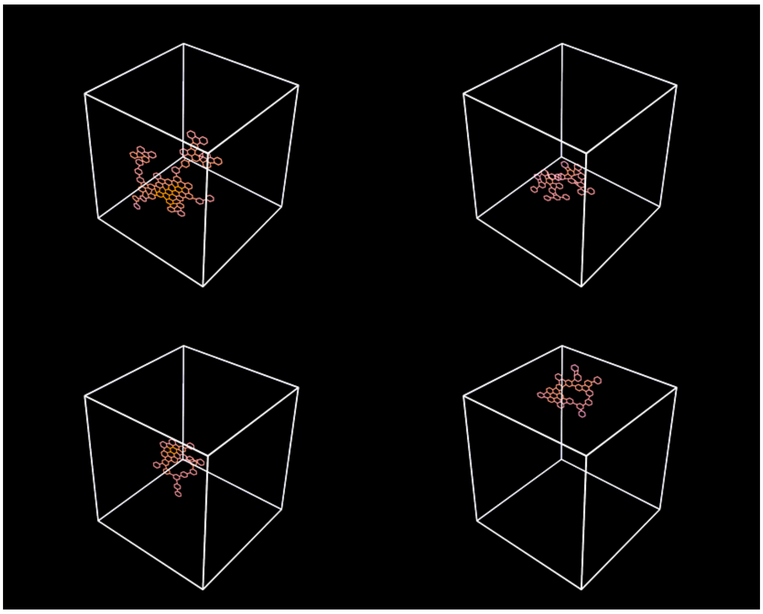

Fig. S5 Graphene units in HRMD simulation of mid-crystalline carbon.

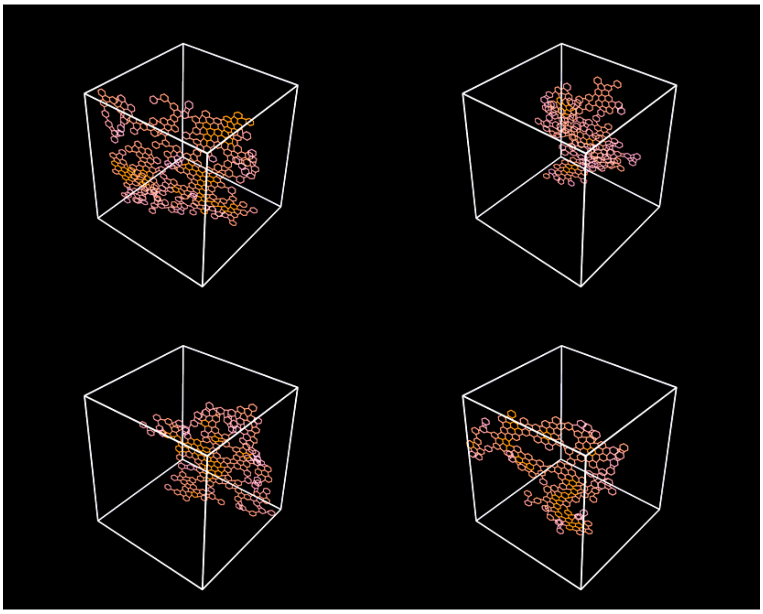

Fig. S6 Graphene units in HRMD simulation of highly-crystalline carbon.
